# Basic Susceptibility of Patients with Psoriasis under Systemic Therapy for Respiratory Infections: Data from the German Psoriasis Registry PsoBest

**DOI:** 10.3390/jcm13133713

**Published:** 2024-06-26

**Authors:** Brigitte Stephan, Stephan Jeff Rustenbach, Nesrine Ben-Anaya, Matthias Augustin, Wolf-Henning Boehncke, Michael Hertl, Ulrich Mrowietz, Petra Staubach-Renz, Diamant Thaçi, Ralph von Kiedrowski, Christina Sorbe

**Affiliations:** 1 Institute for Health Services Research in Dermatology and Nursing (IVDP), University Medical Center Hamburg-Eppendorf (UKE), 20246 Hamburg, Germany; s.rustenbach@uke.de (S.J.R.); n.ben-anaya@uke.de (N.B.-A.); m.augustin@uke.de (M.A.); c.sorbe@uke.de (C.S.); 2 Department of Dermatology and Venereology, Geneva University Hospitals, 1205 Geneva, Switzerland; wolf-henning.boehncke@hcuge.ch; 3 Department of Dermatology, University Hospital Marburg, 35043 Marburg, Germany; hertl@med.uni-marburg.de; 4 Department of Dermatology, University Medical Center Schleswig-Holstein, Campus Kiel, 24105 Kiel, Germany; umrowietz@dermatology.uni-kiel.de; 5 Department of Dermatology, University Medical Center Mainz, 55131 Mainz, Germany; petra.staubach@unimedizin-mainz.de; 6 Comprehensive Center for Inflammation Medicine, University Hospital Schleswig-Holstein, Campus Lübeck, 23562 Lübeck, Germany; diamant.thaci@uksh.de; 7 Dermatological Practice, 56242 Selters, Germany; dr.ralph.vonkiedrowski@praxis-kiedrowski.de

**Keywords:** biologics, pre-COVID, psoriasis, respiratory infections

## Abstract

**Background**: Patients with psoriasis under systemic treatments are in focus regarding their susceptibility to respiratory infections. To analyse real-world data for respiratory infections in patients with psoriasis under systemic treatments. **Methods**: We analysed data of the prospective, non-interventional German Psoriasis Registry PsoBest and compared rates for respiratory infections of 13,823 patients on systemic treatments for psoriasis and/or psoriatic arthritis in different therapy cohorts before the COVID-19 pandemic. **Results**: In total, 1415 respiratory infections were observed in 970 patients. Significant differences were observed between biologics and non-biologics, but not within these groups. The highest event rates (events/100 patient years) were identified for TNF-α inhibitors, 8.1, (CI 7.4–8.9), followed by 7.0 for IL-17 inhibitors (6.2–7.9), 5.7 for IL-12/23 and IL-23 inhibitors (5.1–6.5), 4.8 for methotrexate (4.3–5.4), 3.7 for small molecules (3.3–4.2), and 2.7 for retinoids (1.2–5.1). **Conclusions**: Overall, the susceptibility for respiratory infections in patients under systemic therapy for psoriasis is low compared to published study data and is sufficient as comparative data for COVID-19 studies.

## 1. Introduction

With the recent pandemic, the susceptibility for respiratory infections in patients with psoriasis under systemic treatments has become significantly important. With the rapid spreading of corona cases all over the world in the spring of 2020, medical societies tried to analyse the risk for respiratory infections in special patient groups and communicated cautious recommendations based on acute observations and experiences regarding continuing systemic medications during acute infection [1,2,3,4,5]. Patients with comorbidities, like obesity, diabetes, cardiovascular diseases, asthma, or chronic obstructive pulmonary disease (COPD), were seen to be at increased risk for infections [6,7,8,9,10,11]. With the proceeding pandemic and increasing registered cases, medical specialties relativised the estimated susceptibility for SARS-CoV2 infections in patients under systemic medications, and at the beginning of 2021, vaccinations were available and strongly recommended for them to be kept up to date [12,13,14,15,16,17,18].

Respiratory infections are of key interest in randomised controlled trials (RCTs) for systemic medications and especially with the advent of novel therapeutic groups of biologics and Janus kinase (JAK) inhibitors, there is a major focus on these adverse events. It is a matter of debate as to whether these therapeutics themselves or the underlying medical conditions treated cause susceptibility to infections and if the patients preselected for studies depict the real risk of patients with psoriasis or psoriatic arthritis under systemic therapies. In contrast, RCT data from registries provide real-world evidence about the risks patients face in daily treatment and may contribute critical information in this regard.

We compared rates of respiratory infections in patients on systemic treatment for psoriasis in different therapy cohorts using data from the German Psoriasis Registry PsoBest before the COVID-19 pandemic. This registry observes psoriasis patients with or without psoriatic arthritis who underwent systemic treatment for these two indications, which reflect at least moderate severity of the disease. We focused on the prevalence rates of respiratory infections before the SARS-CoV2 pandemic reached Germany in February 2020 [19,20] to analyse data before the changes in the conduct of respiratory events as a reference baseline for further analyses of data during the pandemic.

## 2. Materials and Methods

### 2.1. Registry and Patients

Since 2008, the German Psoriasis Registry PsoBest has included adults with from moderate to severe psoriasis with or without psoriatic arthritis. Data are being collected at about 1100 dermatological offices and outpatient clinics. Dermatologists and patients fill out standardised questionnaires on disease conditions, treatments, and adverse events, as well as patient-reported outcomes on quality of life and disease burden. Patients who gave informed consent are eligible for inclusion at the start of a systemic treatment. They are observed at the baseline; months 3, 6, 9, and 12 after the treatment starts; and every 6 months afterwards for up to 10 years. Within this time, treatment changes are allowed.

This analysis comprises all the quality-ensured data collected until December 2021. Despite the documentation of several other treatment options, we focused on the most frequently used ones: tumour necrosis factor (TNF) inhibitors (adalimumab, certolizumab, etanercept, golimumab, and infliximab, including biosimilars), interleukin (IL)-17 inhibitors (brodalumab, secukinumab, and ixekizumab), IL-12/23 and IL-23 inhibitors (guselkumab, ustekinumab, tildrakizumab, and risankizumab), as well as small molecules (apremilast, ciclosporin, fumaric acid esters, tofacitinib, methotrexate, and retinoids). Patients who received at least one of these treatments for at least one day were included in this analysis.

### 2.2. Events

Adverse events and serious adverse events were reported to the registry and coded in preferred terms, following the Medical Dictionary for Regulatory Activities (MedDRA^®^), including system organ classes (SOCs), which allow for the classification of events in general.

For this analysis, all the MedDRA event codes with primary or secondary SOCs “Infections and Infestations” were screened for respiratory events, resulting in a list of potential respiratory tract infections, including, e.g., bronchitis, pneumonia, and influenza (supplemental Table S1).

A safety board reviewed all the events, which met at least one of these criteria, reported to PsoBest and classified them into serious and non-serious cases according to current guidelines on clinical safety data management [21]. Diseases other than respiratory infections were not a part of this analysis. Events were assigned to a treatment if the onset of the events was between the treatment start and stop date plus a 90-day risk window. If an event met more than one treatment criterion, it was assigned to all the appropriate treatments but counted only once in pooled groups. When calculating the total therapy times for the rates per patient years, the durations of all the therapies were again taken into account. If infections were reported by December 2021 but started before February 2020, we excluded them from the analysis to exclude the effects of the COVID-19 pandemic.

### 2.3. Statistics

Descriptive statistics of this as-observed analysis were performed using standard parameters: absolute and relative frequencies for categorical data and minimum, median, maximum, and standard deviation (SD) for continuous data.

We present data from the registry baseline for all the patients in the respective treatment groups. Therefore, patients who received more than one treatment within the registry are included in more than one baseline set, which is not necessarily a treatment baseline. For safety analysis, we calculated standardised event rates per 100 patient years (py) as well as 95% confidence intervals using an inverse chi-squared distribution. Significant differences between groups were detected using the interval method at a significance level of 0.05. Subgroup analyses were performed for patients’ baseline characteristics: by sex (male vs. female), by age (18–34 vs. 35–65 vs. 66+ years), by previous treatment (no prior systemic treatment vs. systemic but no biological pre-treatment vs. biological pre-treatment), by body mass index (BMI, BMI < 18.5 (underweight) vs. BMI 18.5–24.9 (normal weight) vs. BMI 25–29.9 (overweight) vs. BMI 30–34.9 (obese, class I) vs. BMI 35–39.9 (obese, class II) vs. BMI ≥ 40 (obese, class III), and by comorbidity (0–2 vs. 3+ different comorbidity groups). Patients with three or more different comorbidity groups are referred to as multimorbid. 

There were neither imputations for missing values nor tests of the hypothesis because this is an exploratory research approach. The statistical analysis was conducted using SPSS v. 27 (IBM, Armonk, NY, USA).

## 3. Results

The 12,836 patients analysed were included between 2008 and 2021. They were predominately male (58.4%), with median ages of 48.0 years (18.0–93.0) and 14.7 at the registry entry. The median disease duration was 14.0 (0.0–76.0) years. A total of 47.7% showed nail involvement, and 31.1% showed joint involvement. The median psoriasis area and severity index (PASI) was 13 (0–72), and the median involved body surface area (BSA) was 18.0 (0–100). With a median dermatology life quality index (DLQI) of 11.0 (0–30), patients showed a marked burden of the disease. Patients in the registry also show high rates of comorbidities with, e.g., obesity/adipositas or diabetes [22], and our patients had median BMIs of 28.1 (14.2–61.1) in the group treated with biologics and 27.6 (14.7–67.3) in the non-biologics group.

The majority of the patients received non-biological treatments in their “registry life” (*n* = 8136, 63.4%). Among those, fumaric acid esters (*n* = 4024, 31.3%) were the most common, followed by methotrexate (*n* = 3826, 29.8%). Apremilast, ciclosporin, and retinoids were tried in less than 1000 patients. Among the biologics, which were observed in a total of *n* = 6538 patients (50.9%), patients most frequently received IL-12/23 and IL-23 inhibitors (*n* = 2594, 20.2%) and IL-17 inhibitors (*n* = 2525, 19.7%), followed by TNF inhibitors (*n* = 2388, 18.6%).

Table 1 and Table 2 show the number of patients exposed to both biological and non-biological treatments, as well as patients’ characteristics, at the registry baseline concerning the treatment received. 

We found significant differences between patients with biological and non-biological treatments: patients receiving biological treatments within their registry life are more likely to be male (56.8% vs. 40.9%, *p* < 0.05), have nail psoriasis (52.8% vs. 45.1%, *p* < 0.05) or psoriatic arthritis (40.3% vs. 27.2%, *p* < 0.05), and tend to be multimorbid (11.4% vs. 8.9%, *p* < 0.05). Furthermore, patients with biological treatments tend to have higher PASIs (16.1 and 14.3, *p* ≥ 0.05), BSAs (25.7 and 23.1, *p* ≥ 0.05), and DLQIs (12.5 and 11.1, *p* ≥ 0.05).

With the focus on respiratory infections, in total, 1415 respiratory infections in 970 patients were reported in 6538 cases with biologics (13,841 py) and 8136 patients receiving non-biological treatments (13,648 py).

**Figure 1:**
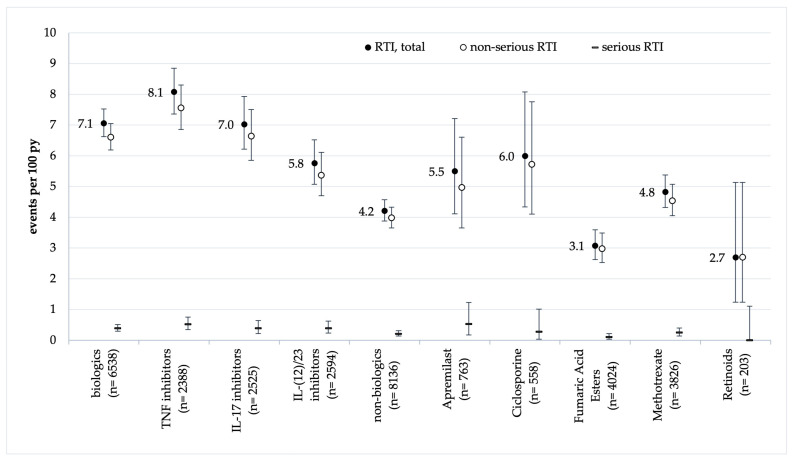
Rates of respiratory tract infections (RTIs) by treatment and event severity. Vertical bars indicate 95% confidence intervals.

The highest event rates of respiratory tract infections (RTIs) were identified for TNF-α inhibitors (8.1 events/100 py, 95% CI 7.4–8.9), followed by 7.0 for IL-17 inhibitors (6.2–7.9), 5.8 for IL-12/23 and IL-23 inhibitors (5.1–6.5), 4.8 for methotrexate (4.3–5.4), 3.7 for small molecules (3.3–4.2), and 2.7 for retinoids (1.2–5.1, Figure 1 and Table S2). Event rates of serious RTIs were below 1 event per 100 py in all the treatment groups and did not differ significantly.

Statistically significant differences were identified between biological and non-biological treatments (7.1 vs. 4.2 RTI/100 py, *p* < 0.05). Within the group of biological treatments, we found significantly lower rates of RTIs in IL-12/23 and IL-23 inhibitors compared to TNF-α inhibitors: 5.8 vs. 8.1 infections/100 py (*p* < 0.05). Within non-biologics, there were no significant differences.

Although the patients on biologics generally showed higher rates of RTIs, we found, again, increased rates in female patients (8.6 vs. 6.1 on biologics and 4.9 vs. 3.7 on non-biologics, *p* < 0.05), in younger patients (8.1 in patients aged 18–34 years vs. 7.1 and 4.5 in patients aged 35–65 and 66+ on biologics, *p* < 0.05), and in multimorbid patients (9.0 vs. 6.8 on biologics, *p* < 0.05). Previous treatments as well as BMI at the registry baseline did not show any association with RTI rates.

## 4. Discussion

Safety data from RCTs and especially real-world data on the long-term use of medications can add helpful information about the risk for infections under therapy.

Systemic corticosteroids have been the first drugs of choice for many decades, showing a potent downregulation with immediate effects on chronic inflammation, but their severe side effects in long-term use together with better options for targeted therapies led to a change in recommendations avoiding long-term use [23,24].

Conventional systemic treatments, like methotrexate, are frequently used in dermatology [25], but safety studies for use in chronic inflammatory diseases, like psoriasis, and registry data [26] did not show increased risk signals for overall infections under therapy [22,27,28,29]. This correlates with data from randomised controlled trials among patients under low-dose therapy with methotrexate, confirming slight increases in pneumonia as well as in upper respiratory infections, which, however, were not significant [30].

Our analyses focus especially on respiratory infections, and we found low rates, with 3.05 (2.76–3.36) patients with non-serious infections/100 py in non-biological treatments and 4.41 (4.07–4.78) in biologics as well as 0.23 (0.16–0.33) patients with serious respiratory tract infection(s) in non-biologics and 0.45 (0.34–0.57) in biological treatments (Table S2).

Ciclosporin, a potent immunosuppressor at higher doses and approved for use in severe psoriasis at lower doses in all the EU countries [31,32,33,34,35,36,37,38,39,40], shows higher rates for infections compared to other systemic medications, like methotrexate [29]. In comparison with our analysis, we found a rate of 6.00 (95% CI 4.34–8.08) respiratory infections/100 py under treatment (as-exposed analysis) with ciclosporin.

The retinoid acitretin has been used for psoriasis [41,42] for more than 40 years [43,44,45], and its overall infection rates do not pose a special issue [23,29,46,47]. Data from our registry reflect a low rate of 2.70 (1.24–5.13) events/100 py for non-serious and no documented serious events for respiratory infections.

Dimethyl fumarate does not show elevated rates for respiratory infections in the treatment of psoriasis [48]. Fumarates have also been approved for the treatment of multiple sclerosis since 2013, and in concordance with observations for psoriasis treatment, clinical studies for efficacy and safety in 2012 confirmed no increased risks for respiratory events [49,50]. In our registry, we could confirm a low rate of 3.08 (2.62–3.59) respiratory infections/100 py.

A new approach for the systemic therapy of psoriasis is derived from experiences of rheumatology in treating autoimmune inflammation of the joints with biologics. The first group available for use in psoriasis was the TNF-α inhibitors, and several studies showed data on risks and safety [51,52,53,54,55], with one of the frequently mentioned findings of increased rates of opportunistic infections, like respiratory infections [53]. Our registry data show an event rate of 7.56 (6.86–8.3)/100 py for non-serious and 0.52 (0.35–0.75) events/100 py for serious respiratory infections under treatment with TNF-α inhibitors. Compared to non-biological treatments (3.98 [3.65–4.33] non-serious and 0.21 [0.14–0.31] serious events/100 py), this means a 1.9-fold higher risk for non-serious and 2.5-fold higher risk for serious infections with TNF-α inhibitors versus non-biological treatments (Table S2).

A biologic targeting the IL-12/23 inhibitor and approved for the treatment of psoriasis or psoriatic arthritis is ustekinumab [56]. Data from the observational study PSOLAR (psoriasis longitudinal assessment and registry), recruiting since 2007, have revealed that more than 16,000 participants indicated lower rates for serious adverse events, including respiratory events, compared to the rates under treatment with TNF-α inhibitors [57]. IL-23 inhibitors showed respiratory infections as the most common adverse events but overall low rates in long-term safety data from randomised controlled trials [58,59,60,61]. We could find rates of 5.37 (4.70–6.11) non-serious events/100 py and 0.39 (0.23–0.62) serious events/100 py for IL-12/23 and IL-23 inhibitors (including ustekinumab) (Figure 1 and Table S2), which signify a 1.4-fold higher rate compared to non-biological treatments but not differing significantly between IL-23 inhibitors themselves (Figure 1 and Table S2).

For IL17 inhibitors, the most frequently reported adverse events in approval studies were nasopharyngitis, oral candidiasis, and upper respiratory tract infections, mostly from mild to moderate in severity without discontinuation of the study treatment (EMA summary product characteristics) [62,63,64,65]. In follow-up studies on long-term use, a consistent safety profile could be confirmed. Data from our registry show rates of 6.64 (5.85–7.51) non-serious respiratory infections/100 py and 0.39 (0.22–0.64) serious events/100 py. This means a 1.7-fold higher rate for non-serious events and a 1.9-fold higher rate for serious respiratory infections compared to non-biological treatments. Within a total of 7.03 (6.22–7.93) respiratory infections/100 py, this indicates a 1.7-fold higher rate for IL-17 inhibitors than for non-biological treatments, a 1.2-fold higher rate for IL-23/12/23 inhibitors, and a 0.9-fold higher rate compared to TNF-α inhibitors (Figure 1 and Table S2).

With the phosphodiesterase inhibitor apremilast, the most frequently reported adverse events were gastrointestinal disorders with diarrhoea or nausea. Safety studies reported rates for upper respiratory infections, with 14.7/100 py and 28.8/100 py (exposure adjusted), mostly from mild to moderate severity [56,66]. Our data reflect a rate of 5.50 (4.11–7.21) respiratory infections/100 py, comparable to infection rates with methotrexate (Figure 1 and Table 2).

Studies on opportunistic infections under systemic therapy with JAK inhibitors are published for patients treated for ulcerative colitis or rheumatoid arthritis [67,68] or psoriatic arthritis [69]. Because of the lack of a sufficient number of patient years for a robust analysis, we postponed the analysis of these data for a later publication. Interestingly, another JAK inhibitor (baricitinib), which has no approval for psoriasis or psoriatic arthritis to date, received an emergency use authorisation by the US federal drug agency (FDA) for the treatment of children from age 2 and adults with severe COVID-19 infection and need for oxygen and mechanical ventilation or extracorporeal membrane oxygenation [70]. The use of baricitinib supports the control of the inflammatory process and cytokine release. Its anti-inflammatory potential for respiratory processes will be the subject of further investigation and of interest for the group of chronic inflammatory skin diseases.

The higher RTI rates observed in biological treatments are clearly related to non-serious events. Event rates of serious RTIs were below 1 event per 100 py in all the treatment groups and did not differ significantly.

Significant differences were identified between biological and non-biological treatments (7.1 vs. 4.2 RTI/100 py, *p* < 0.05). Within the group of biological treatments, we found significantly lower rates of RTIs for IL-12 and IL-12/23 inhibitors compared to TNF-α inhibitors: 5.8 vs. 8.1 infections/100 py (*p* < 0.05). Within non-biologics, there were no significant differences.

Focusing on subgroups, which are seen as being at an increased risk for respiratory infections, we found a significantly higher rate for respiratory infections with multimorbidity, as expected. Interestingly, older patients aged 66+, who are expected to be increasingly susceptible to respiratory infections, showed significantly lower event rates for respiratory infections under systemic treatments with biologics compared to the younger age groups. 

If we compare our findings with data from RCTs, we find lower rates for infections. For example, placebo controls, in the RCT reported by Yui et al., represent patients with psoriasis without systemic treatment. The authors found a rate of serious infections, i.e., other than respiratory infections, of 0.4% of patients. In our data, we found 0.1–1.1% (depending on the treatment) only for serious respiratory infections, which can be explained by the highly selective nature of clinical trials [71]. If we focus on patient rates, given by data from the RKI, 6.0% of persons in the average German population were reported with respiratory tract infections in January 2020 [72]; in our registry data, we saw a rate of 7.6%, also indicating a slightly increased susceptibility of patients with psoriasis to respiratory infections. Additionally, we know from other real-world data sources that patients with psoriasis have a slightly increased risk of respiratory infections as such [73].

## 5. Limitations

As in all registries collecting data from real-world treatments, there is underreporting of non-serious medical conditions in daily life, especially for respiratory tract infections that do not require major medical intervention and are, thus, neglected. At the time of the analysis, in our registry, there were too few robust data available for new medications because of a lack of long-term use (e.g., Janus kinase inhibitors or bimekizumab). 

Furthermore, correlated error terms cannot be excluded by considering patient-specific exposure times after the last infection. Together with the potential multiple counting of patients, this leads to rather wider confidence intervals so that actual differences between the treatment groups might be detected rather late.

## 6. Conclusions

The data from this large-scale cohort demonstrate differences between the systemic medication groups regarding the rates for respiratory infections under systemic treatment. They are sufficient as comparative data for COVID-19 studies. Overall, the susceptibility for respiratory infections under systemic therapy for psoriasis is low compared to published study data.

A further analysis is in progress with the data received during the ongoing pandemic with SARS-CoV-2 to gain information about the susceptibility of our patients to this respiratory infection under systemic treatments for psoriasis or psoriatic arthritis.

## Figures and Tables

**Table 1:** Patient characteristics at the registry baseline and exposure years in biological treatments.

	Biologics, Total		TNF Inhibitors	
	6538 Patients		2388 Patients	
	13,841 Patient Years (py)		5745 py	
	Mean (SD)	Median (Range)	Mean (SD)	Median (Range)
Female sex, *n* (%)	2534 (39.1)		987 (41.3)	
Age, years	47.5 (14.1)	48.0 (18.0–93.0)	46.7 (13.8)	47.0 (18.0–87.0)
Body mass index (BMI), kg/m^2^	29.1 (6.1)	28.1 (14.2–61.1)	29.0 (6.0)	28.1 (15.8–57.9)
Duration of psoriasis, years	19.5 (14.4)	17.0 (0.0–74.0)	18.6 (13.9)	16.0 (0.0–68.0)
Nail psoriasis, *n* (%)	3438 (52.8) **		1319 (55.2)	
Psoriatic arthritis, *n* (%)	2620 (40.3) **		1208 (50.6)	
Multimorbid *, *n* (%)	747 (11.4) **		251 (10.5)	
Psoriasis area and severity index (PASI)	16.1 (10.8)	14.0 (0.0–72.0)	15.3 (10.7)	13.4 (0.0–70.8)
Body surface area (BSA)	25.7 (21.0)	20.0 (0.0–100.0)	23.9 (20.6)	17.0 (0.0–100.0)
Dermatology life quality index (DLQI)	12.5 (7.5)	12.0 (0.0–30.0)	12.2 (7.5)	12.0 (0.0 –30.0)
	**IL-17 Inhibitors**		**IL-12/23 and IL-23 Inhibitors**	
	**2525 Patients**		**2594 Patients**	
	**3838 Exposure Years**		**4357 Exposure Years**	
	**Mean (SD)**	**Median (Range)**	**Mean (SD)**	**Median (Range)**
Female sex, *n* (%)	988 (39.1)		1006 (38.8)	
Age, years	48.1 (14.0)	49.0 (18.0–89.0)	47.5 (14.4)	48.0 (18.0–93.0)
BMI, kg/m^2^	29.2 (6.0)	28.4 (16.6–61.1)	29.4 (6.2)	28.4 (14.2–60.9)
Duration of psoriasis, years	20.0 (14.7)	18.0 (0.0–74.0)	19.7 (14.2)	17.0 (0.0–70.0)
Nail psoriasis, *n* (%)	1356 (53.7)		1325 (51.1)	
Psoriatic arthritis, *n* (%)	1026 (40.6)		906 (34.9)	
Multimorbid *, *n* (%)	312 (12.4)		306 (11.8)	
PASI	16.6 (10.8)	14.4 (0.0–70.8)	16.3 (10.6)	14.1 (0.0–72.0)
BSA	26.9 (21.6)	20.0 (0.0–100.0)	26.0 (20.5)	20.0 (0.0–98.0)
DLQI	12.9 (7.5)	12.0 (0.0–30.0)	12.4 (7.5)	12.0 (0.0–30.0)

Significant differences (*p* < 0.05) between biologics and non-biologics (Table 2) are marked with **. Other treatment groups were not tested against each other. The number of patients is not additive because patients may have received more than one treatment. * Patients with 3 or more different comorbidity groups are referred to as multimorbid.

**Table 2:** Patient characteristics at the registry baseline and exposure years by treatment.

	Non–Biologics, Total		Apremilast		Ciclosporin	
	8136 Patients		763 Patients		558 Patients	
	13,648 Exposure Years		946 Exposure Years		717 Exposure Years	
	Mean (SD)	Median (Range)	Mean (SD)	Median (Range)	Mean (SD)	Median (Range)
Female sex, *n* (%)	3513 (43.2)		361 (47.3)		255 (45.7)	
Age, years	47.8 (15.0)	48.0 (18.0–92.0)	52.0 (15.6)	52.0 (18.0–89.0)	43.5 (14.4)	43.0 (18.0–92.0)
Body mass index (BMI), kg/m^2^	28.5 (5.9)	27.6 (14.7–67.3)	29.0 (6.2)	28.0 (15.9–60.6)	27.7 (6.0)	26.8 (15.9–63.3)
Duration of psoriasis, years	15.9 (14.5)	12.0 (0.0–76.0)	20.0 (16.0)	17.0 (0.0–76.0)	15.3 (12.8)	12.0 (0.0–68.0)
Nail psoriasis, *n* (%)	3669 (45.1) **		389 (51.0)		274 (49.1)	
Psoriatic arthritis, *n* (%)	2209 (27.2) **		289 (37.9)		149 (26.7)	
Multimorbid *, *n* (%)	727 (8.9) **		120 (15.7)		27 (4.8)	
Psoriasis area and severity index (PASI)	14.3 (9.6)	12.4 (0.0–70.8)	6.5 (2.3)	7.0 (0.0–10.0)	6.9 (2.1)	7.0 (0.0–10.0)
Body surface area (BSA)	23.1 (19.2)	16.0 (0.0–100.0)	21.9 (18.1)	15.0 (0.0–95.5)	23.1 (19.7)	16.0 (0.0–100.0)
Dermatology life quality index (DLQI)	11.1 (7.1)	10.0 (0.0–30.0)	11.1 (7.0)	10.0 (0.0–30.0)	12.1 (7.1)	11.0 (0.0–30.0)
	**Fumaric Acid Esters**		**Methotrexate**		**Retinoids**	
	**4024 Patients**		**3826 Patients**		**203 Patients**	
	**5231 Exposure Years**		**6874 Exposure Years**		**333 Exposure Years**	
	**Mean (SD)**	**Median (Range)**	**Mean (SD)**	**Median (Range)**	**Mean (SD)**	**Median (Range)**
Female sex, *n* (%)	1691 (42.0)		1621 (42.4)		105 (51.7)	
Age, years	45.5 (15.4)	45.0 (18.0–92.0)	49.4 (13.7)	50.0 (18.0–88.0)	53.8 (14.4)	55.0 (18.0–83.0)
BMI, kg/m^2^	28.2 (5.9)	27.2 (14.7–63.0)	28.9 (5.9)	28.0 (15.4–67.3)	28.2 (5.1)	27.7 (17.6–46.3)
Duration of psoriasis, years	14.3 (13.8)	10.0 (0.0–68.0)	17.1 (14.7)	14.0 (0.0–76.0)	13.4 (14.6)	7.0 (0.0–53.0)
Nail psoriasis, *n* (%)	1648 (41.0)		1873 (49.0)		99 (48.8)	
Psoriatic arthritis, *n* (%)	622 (15.5)		1503 (39.3)		38 (18.7)	
Multimorbid *, *n* (%)	286 (7.1)		366 (9.6)		27 (13.3)	
PASI	6.5 (2.2)	7.0 (0.0–10.0)	14.4 (10.2)	12.4 (0.0–70.8)	11.9 (9.5)	10.0 (0.0–56.1)
BSA	23.7 (18.8)	18.0 (0.0–100.0)	22.7 (19.4)	15.0 (0.0–100.0)	19.5 (20.3)	12.0 (1.0–100.0)
DLQI	11.0 (6.9)	10.0 (0.0–30.0)	11.3 (7.2)	11.0 (0.0–30.0)	11.2 (6.9)	11.0 (0.0–30.0)

Significant differences (*p* < 0.05) between non-biologics and biologics (Table 1) are marked with **. Other treatment groups were not tested against each other. The number of patients is not additive because patients may have received more than one treatment. * Patients with 3 or more different comorbidity groups are referred to as multimorbid.

## Data Availability

The raw data supporting the conclusions of this article will be made available by the authors on request.

## Supplementary Materials

The following supporting information can be downloaded at https://www.mdpi.com/article/10.3390/jcm13133713/s1: Table S1: MedDRA-preferred terms referred to as respiratory infections; Table S2. Respiratory tract infections by treatment and event severity.
