# Supplementary material for: Basic Susceptibility of Patients with Psoriasis under Systemic Therapy for Respiratory Infections: Data from the German Psoriasis Registry PsoBest
Source: J Clin Med. 2024 Jun 26;13(13):3713. doi: 10.3390/jcm13133713 (PMC11242749; doi:10.3390/jcm13133713)
Supplement: Supplementary file 1 [file jcm-13-03713-s001.zip › jcm-3012142-supplementary.pdf]

Supplement: Basic Susceptibility of Patients with Psoriasis under Systemic Therapy for Respiratory Infections: Data from the German Psoriasis Registry PsoBest

by

Brigitte Stephan, Stephan Jeff Rustenbach, Nesrine Ben-Anaya, Matthias Augustin, Wolf-Henning Boehncke, Michael Hertl, Ulrich Mrowietz, Petra Staubach-Renz, Diamant Thaçi, Ralph von Kiedrowski, Christina Sorbe

Table S1. MedDRA preferred terms referred to as respiratory infection.

MedDRA code	Preferred term
10006448	Bronchiolitis
10006451	Bronchitis
10015030	Epiglottitis
10015108	Epstein-Barr virus infection
10022000	Influenza
10023874	Laryngitis
10024968	Lower respiratory tract infection
10028810	Nasopharyngitis
10034738	Pertussis
10034835	Pharyngitis
10035664	Pneumonia
10035702	Pneumonia haemophilus
10035737	Pneumonia viral
10037440	Pulmonary tuberculosis
10039083	Rhinitis*
10040047	Sepsis
10040753	Sinusitis
10044008	Tonsillitis
10044013	Tonsillitis streptococcal
10044314	Tracheobronchitis
10046306	Upper respiratory tract infection
10047461	Viral infection
10047482	Viral upper respiratory tract infection
10051739	Pulmonary sepsis
10057869	Pharyngitis bacterial
10061736	Bronchitis bacterial
10062106	Respiratory tract infection viral
10062352	Respiratory tract infection
10069767	H1N1 influenza
10071699	Infectious pleural effusion
10073755	Pneumocystis jirovecii pneumonia
10081155	Tracheobronchitis bacterial

*Rhinitis does not include allergic forms.

Table S2. Respiratory tract infections by treatment and event severity.

	Events/100 patient years (0.95 CI)	
	**non–serious**	**serious**
Biologics, total	6.61 (6.19–7.05)	0.39 (0.29–0.51)
TNF inhibitors	7.56 (6.86–8.30)	0.52 (0.35–0.75)
IL-17 inhibitors	6.64 (5.85–7.51)	0.39 (0.22–0.64)
IL-12/23 and IL-23 inhibitors	5.37 (4.70–6.11)	0.39 (0.23–0.62)
Non-biologics, total	3.98 (3.65–4.33)	0.21 (0.14–0.31)
Small molecules	3.54 (3.11–4.02)	0.18 (0.09–0.31)
Methotrexate	4.54 (4.05–5.07)	0.25 (0.14–0.40)
Retinoids	2.70 (1.24–5.13)	0.00 (0.00–1.11)
